# Quantitative analysis of the bilateral coordination and gait asymmetry using inertial measurement unit-based gait analysis

**DOI:** 10.1371/journal.pone.0222913

**Published:** 2019-10-01

**Authors:** Seung Hwan Han, Chang Oh Kim, Kwang Joon Kim, Jeanhong Jeon, Hsienhao Chang, Eun Seo Kim, Hoon Park

**Affiliations:** 1 Department of Orthopaedic Surgery, Gangnam Severance Hospital, Yonsei University College of Medicine, Seoul, Republic of Korea; 2 Division of Geriatrics, Department of Internal Medicine, Severance Hospital, Yonsei University College of Medicine, Seoul, Republic of Korea; West Virginia University, UNITED STATES

## Abstract

Inertial measurement unit (IMU)-based gait analysis can be used to quantitatively analyze the bilateral coordination and gait asymmetry (GA). The purpose of this study was to investigate changes in bilateral coordination and GA due to gait speed using an IMU based gait analysis and identify spatiotemporal factors affecting bilateral coordination and GA. Eighty healthy adults (40 men and 40 women) participated in the study. The mean age was 26.2 years, and the mean body mass index was 22.8 kg/m^2^. Three different walking speeds (80%, 100%, and 120% of preferred walking speed) on a treadmill were applied for 1 min of continuous level walking using a shoe-type IMU-based gait analysis system. The phase coordination index (PCI) and GA were calculated on three different walking speeds. Several variables (gait speed, height, body mass index, cadence, and step length) were analyzed as possible factors affecting the PCI and GA. Bilateral coordination and GA improved during fast walking (p = 0.005 and p = 0.019, respectively) and deteriorated during slow walking (p<0.001 and p = 0.008, respectively), compared with the participants’ preferred walking speeds. The correlation analysis revealed that PCI was negatively correlated with step length at each walking condition and lower gait speed was negatively correlated with PCI and GA during slow walking. Both bilateral coordination and GA had a negative linear relationship with gait speed, showing an improvement in the fast walking condition and deterioration in the slow walking condition. Step length was the factor associated with the change in the bilateral coordination.

## Introduction

Human gait is a complex and rhythmic alternating movement of arms, legs, and trunk which create forward body movement [1]. Given the human gait is considered almost symmetrical [2], objective assessment of the symmetry of gait is important. Assessment of gait symmetry may have a role in guiding the clinician’s treatment decisions and evaluating the progress of several diseases[2–6]. Gait asymmetry (GA) can be analyzed through the following parameters: step length, swing time, and stance time [2,7]. Bilateral coordination is also an important gait characteristic because healthy subjects show suitable gait coordination by controlling each leg separately [8]. The phase coordination index (PCI), reflecting the bilateral coordination of gait, is a temporal gait measure that quantifies accuracy of the anti-phase coordination and the consistency of the gait pattern [7]. This metric was used to quantify the ability of subjects to coordinate left-right stepping on level ground [7]. GA and PCI have been used as an indicator of different orthopedic diseases or status after surgery [4–6].

Inertial measurement unit (IMU) systems have been proposed as an alternative method for gait analysis[9–11], because these are relatively inexpensive, simple, wearable, and require a relatively small amount of laboratory space, compared with conventional systems such as optical motion capture systems [12,13]. Shank- and foot-attached sensors perform better for gait event (i.e., heel strike and toe off) detection, compared with lower trunk-attached IMU sensors [14]. Several validity studies of foot-positioned IMU systems comparing optical motion capture systems have been performed in groups of healthy subjects [13,15,16]. Mean accuracy was 1.5 cm for stride length, 1.4 cm/s for stride velocity, and 1.9 cm for foot clearance [13]. The resultant linear acceleration, cadence, step length and step time showed excellent agreement (ICC: 0.990–1.000) [15]. The accuracy of detecting the stride number and estimating travelled distance was 92.67–100% [16]. Therefore, foot-positioned IMU systems are considered an easy and reliable method to measure gait events.

It was known that several factors had some effect on the PCI and GA. Some authors have found that gait speed might affect PCI and GA [3,17,18]. Aging is another factor that affects deterioration of bilateral coordination and gait asymmetry [18]. PCI and GA were deteriorated in patients with neuro-degenerative diseases such as Parkinson’s disease and Alzheimer's dementia [3,19]. Considering the shorten stride length and increased cadence in patients with Parkinson’s disease[20], spatiotemporal gait parameters may affect the PCI and GA. However, there were no studies that have investigated other gait parameters that could affect the PCI and GA. A recent study reported that more than 23 strides were required to obtain a reliable, characteristic PCI value [21]. This means that calculating PCI using gait data obtained by ground walking on a short distance may be unreliable. The common system of IMU based gait analysis would be a treadmill and it could collect gait data on more than 23 strides. Additionally, there have been no studies to measure the PCI using a shoe-type IMU-based gait analysis system on the treadmill.

Therefore, the objectives of this study were the following: use a shoe-type IMU-based gait analysis system in healthy participants to examine changes in the bilateral coordination and GA according to gait speed modification and to identify spatiotemporal factors affecting the bilateral coordination and GA.

## Materials and methods

### Participants

Eighty healthy young adult (age 20–30 years) volunteers (40 men and 40 women) participated in this study. The mean (± standard deviation (SD)) values for age, height, and body mass index (BMI) for the entire group of participants were 26.2 ± 4.2 years, 1.66 ± 9.1 m, and 22.8 ± 3.1 kg/m^2^, respectively. Participants were included in the study if they reported that they were healthy and free of any clinically significant co-morbidities likely to affect gait. Participants were excluded if they had clinically significant musculoskeletal, cardiovascular, or respiratory diseases, a history of head trauma or any other neurological disease, major depression, or an uncorrected visual disturbance. This study was conducted following the approval of Institutional Review Board of Gangnam Severance Hospital, Yonsei University College of Medicine (IRB No. 3-2018-0080). Each participant provided informed written consent before being included in the study.

### Gait protocol

**Fig 1:**
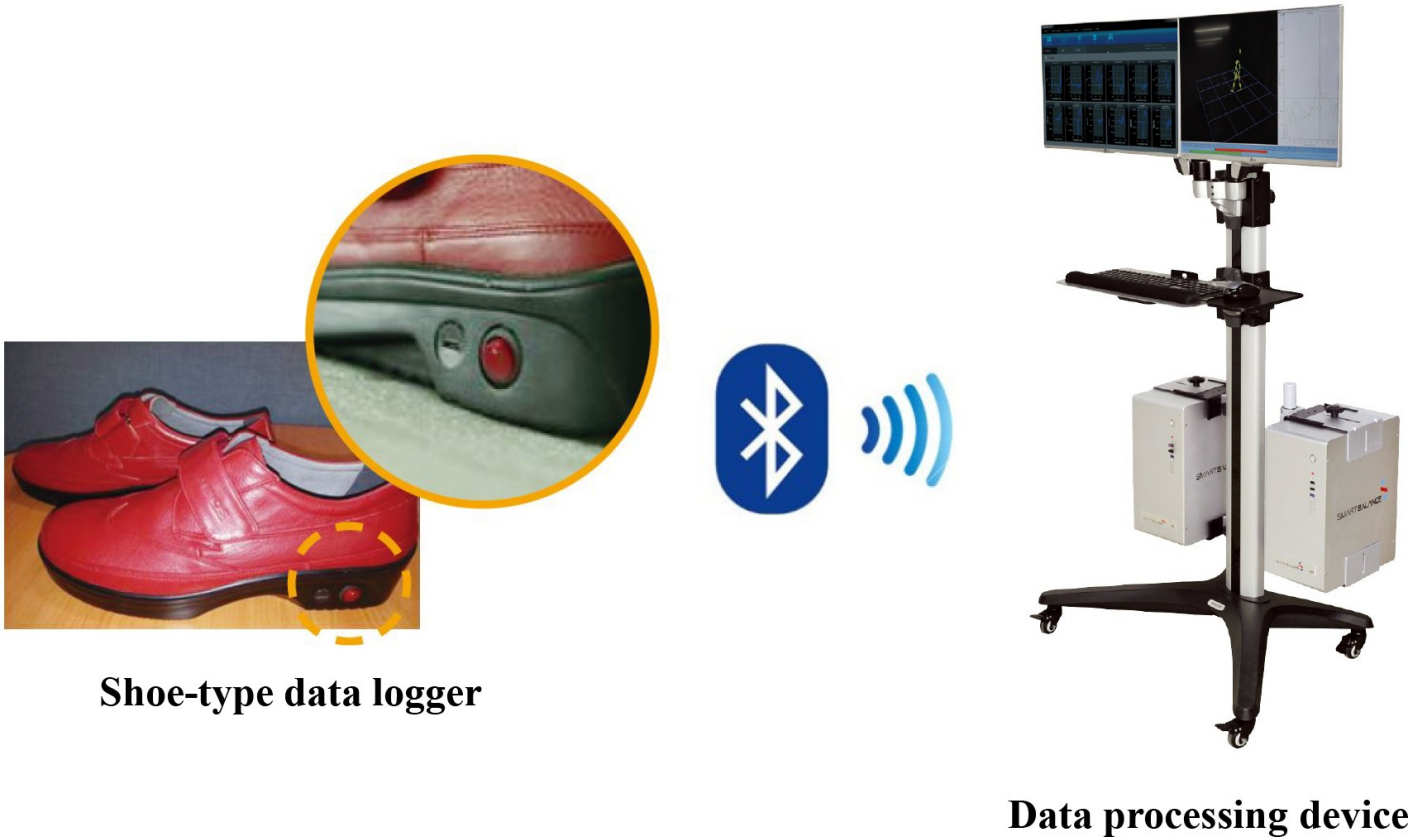
Shoe-type inerial measurement unit (IMU)-based gait analysis system.

An IMU sensor-based gait analysis system (DynaStab^™^, JEIOS, South Korea) consisting of a shoe-type data logger (Smart Balance^®^ SB-1, JEIOS, South Korea) and data acquisition system (DynaStab-Spotfire^®^, Tibco Spotfire 7.10) was used for the study (Fig 1). The shoe-type data logger included an IMU sensor (IMU-3000^™^, InvenSense, USA) that measured tri-axial acceleration (up to ±6 g) and tri-axial angular velocity (up to ± 500° s^−1^) along three orthogonal axes [10, 12]. The IMU sensors were installed in both shoe outsoles, and the data were transmitted wirelessly to a data acquisition system via Bluetooth^®^. The shoe size was adapted to each participant; the range of available shoe sizes was 225 mm to 280 mm. The local coordinate system for the IMU sensors included the anteroposterior, mediolateral, and vertical directions.

All experiments in this study were performed using an inexpensive, commercial feedback-controlled treadmill (RX9200S, TOBEONE, Korea); the belt speed of the treadmill was automatically controlled depending on the location of the participants' body weight with an installed loadcell under the treadmill floor. The speed increased when the walker's location was anterior to the treadmill center and decreased when the walker's location was posterior to the treadmill center [22].

Each participant performed a warm-up protocol comprising a stretching program and 5-min treadmill walking practice with a professional exercise trainer. Each participant was first asked to start walking on the treadmill to find their preferred walking speed. The participants walked for 30–60 s from the start of the gait in order to maintain a steady-state gait. The steady-state gait was defined as that when the participant maintained a stable gait movement at a constant speed [23]. Preferred walking speed was defined as the constant speed at which a participant was able to move with a stable gait while walking on a treadmill. Preferred walking speed was measured by the feedback-controlled treadmill. Then participants were asked to perform three walking trials on the treadmill. The trials were performed at the preferred walking speed (usual walking), and at 80% (slow walking) and 120% (fast walking) of the preferred walking speed. These speeds were typical of those used in other studies [24,25]. Data were collected for 1 min of continuous walking after familiarization with each treadmill speed. A 3-min resting interval between the different speed conditions was used to remove the effects of fatigue.

### Gait analysis

Spatiotemporal parameters were measured using a shoe-type IMU sensor-based gait analysis system. Data on gait speed, cadence, stride and step length, stride and step time, stance phase, swing phase, and double support on each side were collected.

PCI quantifies the bilateral coordination of left-right stepping, following the equations proposed by Plotnik et al.[7]. PCI incorporates the assessment of the accuracy and consistency of phase generation. The step time is used to determine the phase (φ). Normalizing the step time with respect to the stride time, defines the phase of the *i*th stride (φ_*i*_) [7]. We first calculated the mean values of swing times for both legs and used the leg with a higher value of the average swing time as the reference for the gait cycles and calculated phase values for the other leg. Therefore, φ_*i*_ is defined as: φ_*i*_ = 360° x (t_Si_−t_Li_) / (tL(i+1)−t_Li_), where t_Li_ and t_Si_ denote the time of the *i*th heel strike of the legs with the long and short swing times, respectively [7]. The authors considered the phase (φ) as the fluctuation about an ideal line of 180° for each step; φ_ABS was the level of phase generation accuracy and was calculated as the mean value of the series of absolute differences between the phase at each stride and 180°. Therefore, φ_ABS is defined as φ_ABS = | φ_*i*_− 180° |[7]. The CV of the φ mean for each participant (φ_CV) was measured as the level of consistency in phase generation across all of each participant’s strides. Finally, the PCI was calculated as the sum of the two percentile values: PCI = φ_CV + Pφ_ABS, where Pφ_ABS = 100 x (φ_ABS/180)[7]. Lower PCI values indicated a more consistent and more accurate phase generation associated with different health conditions; higher values indicated a more impaired bilateral gait coordination [6].

GA was assessed by comparing the swing times performed by one leg with respect to those performed by the other, following the formula: GA = 100 x | ln(SSWT/LSWT) | where SSWT and LSWT were the mean values of the swing times for the leg with the short and long mean swing time, respectively[2,7]. We calculated the GA using this method because this has been commonly used in previous literature [4,5]. With this definition, values of 0.0 reflect perfect symmetry and high values reflect greater degrees of asymmetry [3].

### Statistical analysis

All continuous variables were tested for normality via Shapiro-Wilk test and did not violate the normal distribution assumption. Gait parameters during fast walking and slow walking were compared with those of usual walking using one-way ANOVA. When significant interaction or main effects were detected between gait speeds, post hoc testing utilizing the Bonferroni correction was performed to make pairwise comparisons among the three groups. Pearson correlation analysis was conducted to examine the relationship between variables, which describes the direction and strength of a relationship between two continuous variables in our study, where the direction can be negative or positive. All statistical analyses were performed using SPSS software. A p-value ≤ 0.05 (two-sided) was considered to indicate a statistically significant result. A post hoc power analysis was also carried out using Power Analysis and Sample Size 11 for Windows software package (NCSS Inc., LLC, Kaysville, UT, USA).

## Results

The results for the mean values for GA, the PCI, and gait speeds during the different walking conditions are presented in Table 1 and S1 File.

**Table 1:** Group mean values of gait parameters at different gait speed.

Gait parameters	Usual walking	Fast walking	Slow walking	p-value
Gait speed (m/s)	1.11 ± 0.05	1.33 ± 0.05	0.88 ± 0.05	
Cadence (steps/min)	111.6 ± 6.5	120.3 ± 6.9	99.7 ± 9.8	<0.001
Step length (m)	0.63 ± 0.08	0.71 ± 0.09	0.56 ± 0.09	<0.001
Swing time (s)	0.43 ± 0.02	0.40 ± 0.02	0.48 ± 0.04	<0.001
Phase coordination index (%)	2.87 ± 0.74	2.65 ± 0.72	3.57 ± 1.05	<0.001
Gait asymmetry (%)	1.27 ± 1.05	0.97 ± 0.87	1.74 ± 1.38	<0.001

We found statistically significant differences for the PCI and GA between the three walking conditions, according to gait speed. The post-hoc test using the Bonferroni correction revealed that the PCI for the usual-walking condition was significantly higher compared with the fast-walking condition (p = 0.005) and was significantly lower compared with the slow-walking condition (p<0.001). The post-hoc analysis also found that GA during the usual-walking condition was significantly higher compared with the fast-walking condition (p = 0.019) and was significantly lower than the slow-walking condition (p = 0.008) (Table 2). Post hoc power analysis revealed that the power was 0.99 for PCI, 0.98 for GA among three groups.

**Table 2:** The results of post hoc analysis between groups according to gait speed.

Gait parameters	Usual vs. Fast (p-value)	Usual vs. Slow (p-value)	Slow vs. Fast (p-value)
Phase coordination index	0.005	<0.001	<0.001
Gait asymmetry	0.019	0.008	<0.001

The results of the correlation analysis are presented in Table 3. The PCI was negatively correlated with only step length on all walking conditions: the usual-walking condition (r = -0.356, p = 0.001), fast-walking condition (r = -0.356, p = 0.001), and slow-walking condition (r = -0.487, p<0.001). Gait speed was inversely associated with the PCI (r = -0.695, p<0.001) and GA speed (r = -0.420, p = 0.023) during only the slow-walking condition. The PCI and GA were not correlated with height, BMI, and cadence on all walking conditions.

**Table 3:** Association between the PCI, GA and other variables at different gait speed.

Variables		Usual walking		Fast walking		Slow walking	
		PCI	GA	PCI	GA	PCI	GA
Gait speed	r	-0.098	-0.206	-0.329	-0.065	**-0.695**	**-0.420**
	*p*-value	(0.613)	(0.285)	(0.082)	(0.739)	**(<0.001)**	**(0.023)**
Height	r	-0.073	0.032	-0.239	-0.089	-0.087	-0.045
	*p*-value	(0.515)	(0.778)	(0.031)	(0.429)	(0.438)	(0.687)
BMI	r	-0.114	0.078	-0.127	0.172	-0.090	0.049
	*p*-value	(0.311)	(0.490)	(0.259)	(0.124)	(0.424)	(0.663)
Cadence	r	0.075	-0.059	0.058	0.102	0.114	-0.171
	*p*-value	(0.504)	(0.599)	(0.607)	(0.366)	(0.309)	(0.128)
Swing time	r	-0.056	0.067	-0.030	-0.102	-0.091	0.185
	*p*-value	(0.621)	(0.550)	(0.788)	(0.367)	(0.421)	(0.098)
Step length	r	**-0.356**	-0.205	**-0.569**	-0.210	**-0.487**	-0.235
	*p*-value	**(0.001)**	(0.067)	**(<0.001)**	(0.060)	**(<0.001)**	(0.053)

Bold means the significant correlation.

## Discussion

We selected the shoe-type IMU system to calculate the PCI and GA for this study because IMU sensors attached bilaterally to each lower limb may increase the detection accuracy of gait sequences such as the heel strike and toe off sequence [12]. The shoe-type IMU system used in our study used sensors mounted in the outsole of the shoe beneath the back of each foot. This arrangement maintained stable sensor positions without hindering movement. This IMU system showed excellent agreement (ICC: 0.990–1.000) with the results for a motion capture system regarding spatiotemporal parameters such as cadence, step time, and step length [15].

Previous studies have calculated the PCI and GA using footswitches, foot pressure insoles, or the GAITRite^®^ system[6,7,17,26,27]. These devices have some weaknesses in several aspects. Although footswitches and foot pressure insoles are wearable and convenient methods for gait variability measurement, they have disadvantages such as limited numbers of detectable gait phases and inappropriate sensor placement[28]. GAITRite^®^ system is commonly used to measure gait sequence and dynamic foot pressure. This system included a 6–13 m long mat containing imbedded electronic pressure-activated sensors [29–31]. Previous studies measuring the PCI used conventional GAITRite^®^ system with 6 m mat [6,27]. The relatively short length of the active area of the GAITRite^®^ system can be a limitation for measurement of averaged stride data. The PCI and GA values may change depending on the numbers of gait cycles included in an analysis. Data from a sufficient number of gait cycles should be collected for accurate measurement of the PCI and GA. Additionally, one study suggested that an average of 22.6 strides were required to obtain reliable and characteristic estimates of PCI values[21]. IMU system can avoid the limitations imposed by the confined spaces of GAITRite^®^ system for measurement of the PCI and GA.

We found a negative linear relationship between PCI and gait speed. This result indicated that gait speed interfered with bilateral coordination; it is not consistent with the results of two other studies[17,18]. One study found that the PCI value was higher in the slow gait condition, compared to usual walking, but was not significantly lower in the fast condition[17]. Their subjects walked back and forth along corridor at a self-determined fast gait speed. We think that self-determined fast gait speed can not be constant while walking back and forth. Another study found that the PCI deteriorated when the participants walk slow and fast[18]. These results may be due to the use of relatively slow gait speeds (0.5–0.9 m/s), compared with normal walking speeds (0.9–1.43 m/s)[32]. Compared with previous studies, our research had its strengths because we included a large number of participants walking at three different gait speeds within the range of normal walking speeds.

There was a negative linear relationship between GA and gait speed. These results are inconsistent with the results of four studies that found that GA is preserved during different gait speeds [17,18,33,34]. Plotnik et al tested 15 healthy young adults on ground walking at their self determined fast and slow speed [17]. Gimmon et al included 13 young adults and asked them to walk on the treadmill at slow gait speed only (0.5~0.9 m/s) [18]. Other studies used GAITRite^®^ system (7 m mat) to measure step length and time asymmetry in healthy young adults [33,34]. In our study, the measurement on the treadmill using IMU system can provide a more constant walking speed and relative longer measurement, compared with the ground walking using GAITRite^®^ system. The more walking cycles are included to calculate the GA, the greater the value of GA. Thus we assumed that different gait protocols and analysis methods might attribute to inconsistent results. Moreover, in previous studies, they did not perform statistic power test in spite of relatively small number of subjects. In this study, we included the 80 healthy subjects and the statistical power is 0.99 for PCI and 0.98 for GA. Nevertheless, further study is need to prove the exact relationship between GA and gait speed because GA was calculated with only swing time in this study.

The correlation analysis for each gait speed condition found that the PCI and GA were independent of gait speed during the usual- and fast-walking conditions. But, during the slow-walking condition, walking more slowly showed deterioration of bilateral coordination and gait symmetricity. These results may reflect how unfamiliar and uncomfortable slow walking makes demands on the attention of participants [11,35]. Cognitive interference may be related with the change of PCI and GA during slow walking [17]. Because fast walking is a common experience during daily living, subjects can walk fast unconsciously. Contrast to fast walking, slow walking is less frequent and subjects are not able to adapt easily and walk rhythmically. Thus, even in healthy adults, unfamiliar slow walking can deteriorate the PCI and GA [17]. Similar results have been found for populations of healthy participants and patients with Parkinson’s disease[7,35].

The correlation analysis found that the shorter the step length, the higher the PCI. Although one study presumed that the PCI might be very weakly associated with stride length[7], our results indicated there was a moderate correlation at every walking condition. These results can explain why the PCI value increases in patients with Parkinson’s disease who have a decreased stride length[20].

This study had several limitations. First, no study has investigated the validity of using IMU-based gait analysis to measure the PCI and GA. It is important to detect correctly the gait event to calculate the PCI and GA. Although only one study proved the validity of shoe-type IMU systems used in our study, they showed an excellent agreement between shoe-type IMU systems and motion capture systems in healthy subjects[15]. Thus, we assume that PCI and GA values are reliable because they were calculated using spatiotemporal parameters. Second, age is a factor that affects changes in the PCI and GA[7,18]. However, we could not investigate the effects of age because the age distribution in our healthy population was too narrow. A third limitation was that our participants walked on a self-controlled speed treadmill, which might have artificially changed gait parameters compared with over-ground walking. However, after a brief training period, the difference between treadmill and over-ground walking is insignificant when walking at a similar speed [36,37]. Additionally, the treadmill walking test may be more useful for collecting steady-state gait data, compared with the over-ground walking test. Treadmill walking is more constrained and differs from over-ground walking [19], which allows acceleration and deceleration of the participants during the gait initiation and termination phases [15]. Therefore, the treadmill walking test is more suitable than the over-ground walking test for quantitative analysis of PCI and GA [38].

In conclusion, both bilateral coordination and GA had a negative linear relationship with gait speed, showing improvement in the fast walking condition and deterioration in the slow walking condition. Step length is a significant factor affecting bilateral coordination of gait.

## Supporting information

S1 File Data tables. (XLSX)
